# Hierarchical effects of pro-inflammatory cytokines on the post-influenza susceptibility to pneumococcal coinfection

**DOI:** 10.1038/srep37045

**Published:** 2016-11-22

**Authors:** Stefanie Duvigneau, Niharika Sharma-Chawla, Alessandro Boianelli, Sabine Stegemann-Koniszewski, Van Kinh Nguyen, Dunja Bruder, Esteban A. Hernandez-Vargas

**Affiliations:** 1 Infection Immunology Group, Institute of Medical Microbiology, Disease Prevention and Control, Otto-von-Guericke University Magdeburg, Germany; 2 Systems Medicine of Infectious Disease Group, Department of Systems Immunology and Braunschweig Integrated Centre of Systems Biology, Helmholtz Centre for Infection Research, Braunschweig, Germany; 3 Immune Regulation Group, Helmholtz Centre for Infection Research, Braunschweig, Germany; 4 Chair for Automation/Modeling, Institute for Automation Engineering, Otto-von-Guericke University Magdeburg, Germany

## Abstract

In the course of influenza A virus (IAV) infections, a secondary bacterial infection frequently leads to serious respiratory conditions provoking high hospitalization and death tolls. Although abundant pro-inflammatory responses have been reported as key contributing factors for these severe dual infections, the relative contributions of cytokines remain largely unclear. In the current study, mathematical modelling based on murine experimental data dissects IFN-*γ* as a cytokine candidate responsible for impaired bacterial clearance, thereby promoting bacterial growth and systemic dissemination during acute IAV infection. We also found a time-dependent detrimental role of IL-6 in curtailing bacterial outgrowth which was not as distinct as for IFN-*γ*. Our numerical simulations suggested a detrimental effect of IFN-*γ* alone and in synergism with IL-6 but no conclusive pathogenic effect of IL-6 and TNF-*α* alone. This work provides a rationale to understand the potential impact of how to manipulate temporal immune components, facilitating the formulation of hypotheses about potential therapeutic strategies to treat coinfections.

Retrospective studies performed on victims of the 1918/1919 influenza A virus (IAV) pandemic and also the recent H1N1 IAV pandemic revealed a high incidence of coinfections with unrelated bacterial pathogens^1^,^2^,^3^,^4^,^5^. In fact, 71% of the high death toll during the 1918/1919 outbreak was attributed to coinfection with *Streptococcus pneumoniae (S. pneumoniae*)^5^. This copathogen is a human adapted gram-positive colonizer of the nasopharynx in asymptomatic children, adults and individuals over 65 years of age but at the same time remains to be the most common cause of community-acquired pneumonia^6^. Animal and human studies have shown that preceding IAV infection enhances all aspects of *S. pneumoniae* pathogenesis from nasopharyngeal colonization to invasive pneumococcal disease^7^, leading to the strong predisposition to lethal secondary pneumococcal infection in IAV infected patients.

Coinfections can be either concurrent or sequential and can involve both acute and chronic infections^8^. Synergistic interactions between pathogens have been well documented for chronic viral infections, for example the influence of prior HIV infection on the development of chronic hepatitis B infection^9^. Regarding IAV-*S. pneumoniae* coinfections, several mechanisms have been implicated in the viral-bacterial synergism which together demonstrated a multifactorial and complex nature of copathogenesis. However, holistic understanding of the effects between IAV, bacteria and immune modulation remain largely unknown.

One central dogma in the viral-bacterial synergism is the disruption of the protective alveolar epithelial cell barrier due to the cytolytic mode of influenza A replication which exposes otherwise cryptic bacterial adherence factors on the basal membrane and thereby promotes invasive pneumococcal disease. More debatable mechanisms are the IAV-mediated immune modulations such as immune cell dysfunction and apoptosis causing an aberrant production of inflammatory mediators in the case of a secondary bacterial encounter. Experimental reports indicate dampened innate inflammatory responses to the bacteria in IAV pre-infected hosts due to an enhanced activation threshold of lung innate immune cells that renders them hypo-responsive^10^. In contrast, a number of studies describe a massive and overshooting inflammatory cell influx due to the hyper-production of pro-inflammatory cytokines such as type I Interferons (IFN-I), Interferon-*γ* (IFN-*γ*), Interleukin-6 (IL-6) and Tumor Necrosis Factor-*α* (TNF-*α*) during secondary bacterial infection. These are often linked to pulmonary edema due to irreparable damage to the alveoli and immunopathology leading to mortality during coinfections^7^,^11^,^12^,^13^,^14^. Taken together, these studies strongly reflect an exacerbated cytokine and chemokine production that may significantly contribute to the detrimental changes in the lung microenvironment that favor secondary bacterial infections. However it is still largely unknown what the relative contribution of these cytokines to bacterial outgrowth, morbidity and mortality in secondary infection is and whether they work alone or in synergism as ‘friend or foe’ to the coinfected host.

Dissecting the detailed contributions of the identified players in enhancing susceptibility to severe secondary bacterial disease following IAV as well as their respective interactions is crucial to develop prophylactic and therapeutic strategies. In the past, mathematical modelling has made valuable contributions to our understanding of IAV infection, focusing either on IAV replication^15^,^16^,^17^,^18^,^19^,^20^,^21^ and/or host immune responses to IAV^22^,^23^,^24^,^25^,^26^,^27^,^28^,^29^,^30^,^31^,^32^. Regarding the interactions between IAV and *S. pneumoniae*, the only modelling approach proposed so far has been through the pioneering work of Smith *et al*.^33^. However, to the best of the authors knowledge, untangling the contributions of the different mechanisms by which changes in the immune response affect bacterial clearance in a temporal manner has not been attempted until now. Therefore, by combining the results of murine *in vivo* experiments and mathematical modelling approaches, we aimed at clarifying the relative contributions of different underlying mechanisms of the IAV-*S. pneumoniae* synergism.

## Results

### Study Design

**Figure 1:**
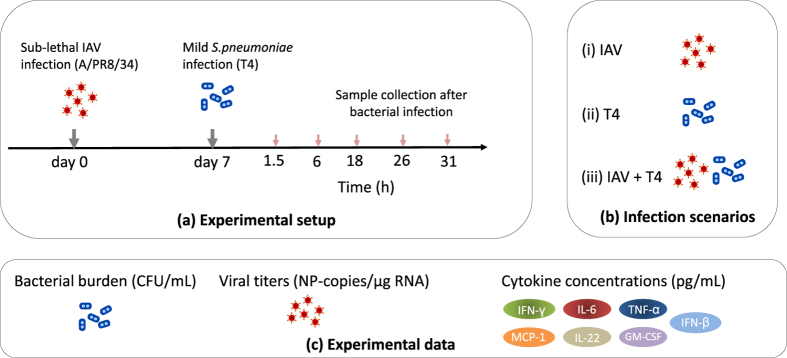
Experimental scheme. (**a**) C57BL/6 J wildtype mice were intranasally infected with a sub-lethal dose of IAV (A/PR8/34) followed by bacterial infection with the *S. pneumoniae* strain T4 on day 7. Bronchoalveolar lavage (BAL), post-lavage lung and blood were collected at the indicated time points post secondary bacterial infection (hpi). (**b**) The infection groups were single viral infection (IAV), single bacterial infection (T4) and coinfection (IAV+T4). (**c**) The bacterial burden, viral titers and cytokine concentrations were determined as the experimental readouts.

The dynamics of IAV and *S. pneumoniae* coinfection were investigated by establishing a murine model displaying disease upon subsequent infection with sub-lethal infection doses of both copathogens. Secondary infection with 1 × 10^6^ colony forming units (CFU) of *Streptococcus pneumoniae* strain TIGR4 (T4) was performed on day 7 after IAV infection based on previous experimental observations that indicated peak susceptibility to pneumococcal disease at this time point during acute IAV infection^3^,^34^. Bacterial burden, viral titers, cytokine concentrations and alveolar macrophage (AM) counts were determined in the respiratory tract for three experimental groups: coinfected (IAV + T4), single IAV and single T4 infected animals. A schematic representation of the experiments is provided in Fig. 1.

The complexity and at times redundancy of immune responses to infections often render to arduous and expensive experimental settings when attempting to identify the key components and their temporal contributions during coinfections. Thus, merging mathematical modelling with the relevant *in vivo* data is a promising tool to unravel complex interactions^25^,^31^,^33^,^35^. In order to dissect the dynamics observed in our experiments, mathematical modelling was employed not as a quantitative recapitulation of experimental data but as a tool to evaluate various hypotheses on the basis of mathematical models and the corrected Akaike information criterion (AICc) for the model selection process.

### Bacterial growth kinetics during IAV-pneumococcal coinfection

**Figure 2:**
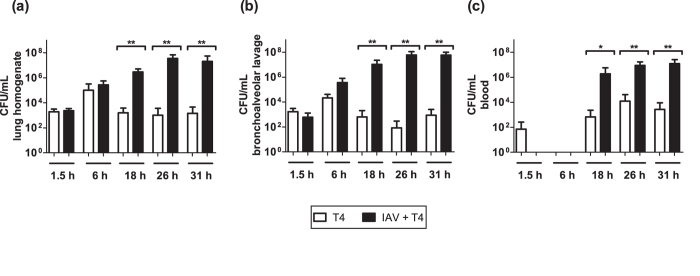
Organ-wide bacterial burden in the single and coinfected animals. Bacterial titers in single T4 infected and IAV+T4 coinfected mice were determined in (**a**) post-lavage lung, (**b**) BAL and (**c**) blood. All experiments were performed in groups of 4–7 WT C57BL/6 J mice, raw data can be found in the Supplementary Fig. S1. Statistical analysis was performed using the Mann-Whitney test. Asterisks indicate significant differences between single and coinfected mice: *p < 0.05; **p < 0.01.

Mouse coinfection experiments revealed that the bacterial load in the post-lavage lung tissue and bronchoalveolar lavage (BAL) was comparable between the coinfected (IAV+T4) and single T4 infected groups until 6 hours post infection (hpi) (Fig. 2a and b). At 18 hpi, a significantly higher bacterial load was observed in both the lung tissue and BAL of the coinfected compared to the single T4 infected group. The bacterial load further increased until 31 hpi in the coinfected group. At the later time points, the high bacterial numbers in the respiratory tract were accompanied by the systemic spread via the blood (bacteremia) in all the coinfected animals (Fig. 2c). Significantly lower grade bacteremia was observed in the single T4 infected mice starting 18 hpi (Fig. 2c).

Taken together, assessing the kinetics of bacterial growth and clearance in the respiratory tract and blood following IAV-S. pneumoniae coinfection revealed a “turning-point” between 6 and 18 hpi. At 18 hpi bacterial outgrowth became clearly evident in the coinfected group and was in strong contrast to the onset of bacterial clearance in the T4 only infected group (also see CFU data for individual mice in Supplementary Fig. S1).

### Absolute AM numbers in the lung homogenate of the coinfected animals may not determine bacterial outgrowth

**Figure 3:**
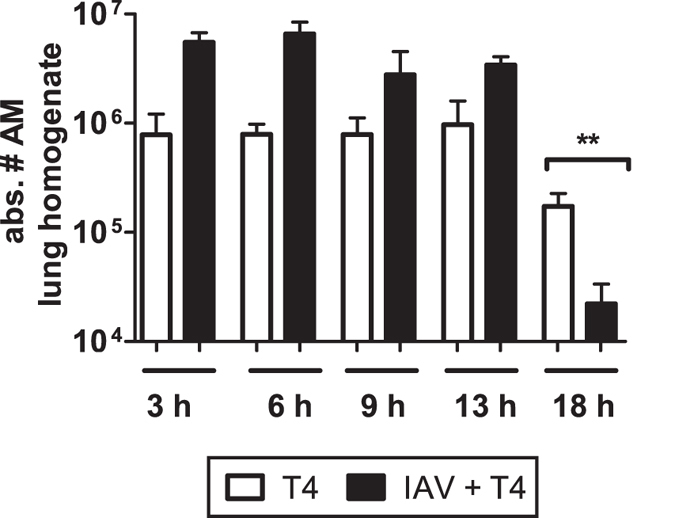
Absolute numbers of alveolar macrophages (AM) in post-lavage lungs of single and coinfected animals. Statistical analysis was performed using the Mann-Whitney test. Asterisks indicate significant differences between single and coinfected mice: **p < 0.01. Experiments were performed in groups of 3–4 WT C57BL/6 J mice.

Previous results in ref. 36 revealed that 90% of resident AMs were depleted in the first week after influenza infection forming a favourable niche for a secondary pneumococcal infection. In contrast, we did not observe IAV-induced AM depletion in our mouse studies but detected significant AM depletion 18 hours post coinfection^37^. To further evaluate dynamic AM numbers in our experimental setting and mathematical modelling approach, we determined the absolute numbers of AMs in the lung tissue at several time-points early after secondary *S. pneumoniae* infection. Experimental results in Fig. 3 show high numbers of AMs in the lung following coinfection that start decreasing at 18 hpi. At this time point, a significant reduction of AMs was detectable in the coinfected animals compared to the bacterial infection alone, which coincided with the established bacterial outgrowth in these mice.

Thus, we evaluated the hypothesis of AM dynamics as a determinant to represent the bacterial kinetics in the single *S. pneumoniae* infected or in the coinfected group. To this end, by using AM count dynamics for the different groups, we proposed several mathematical models to represent the dynamics observed in the single *S. pneumoniae* infected (Supplementary 2) and coinfected animals (Supplementary 3). A model selection process by AIC revealed that assuming bacterial clearance with a constant number of functional AMs (model D2 or D3 at Supplementary 2) provided the best fitting to the data in the single T4 group. In contrast, considering dynamic AM numbers resulted in the worst fitting among the different models for both the single T4 group and the coinfection group. Thereby our model selection study may not support the hypothesis that bacterial clearance is mainly driven by the absolute AM numbers. More likely, only a fraction of functional AMs is required to clear the bacterial infection. Nevertheless, we assume constant AM numbers after 18 hpi for the model M1, while other models in Table 1 are based on experimental data till 31 hpi. Thus, numerical results of the model M1 may need to be taken with caution. Further experiments in mice dissecting functional and non-functional AMs will help to fully determine the effects on the bacterial clearance. To include the experimental data variability in the mathematical model M1, bootstrapping procedures are presented in Supplementary 4.

### Coinfection leads to a significant increase of IFN-*γ*, IL-6 and TNF-*α* airway concentrations

**Figure 4:**
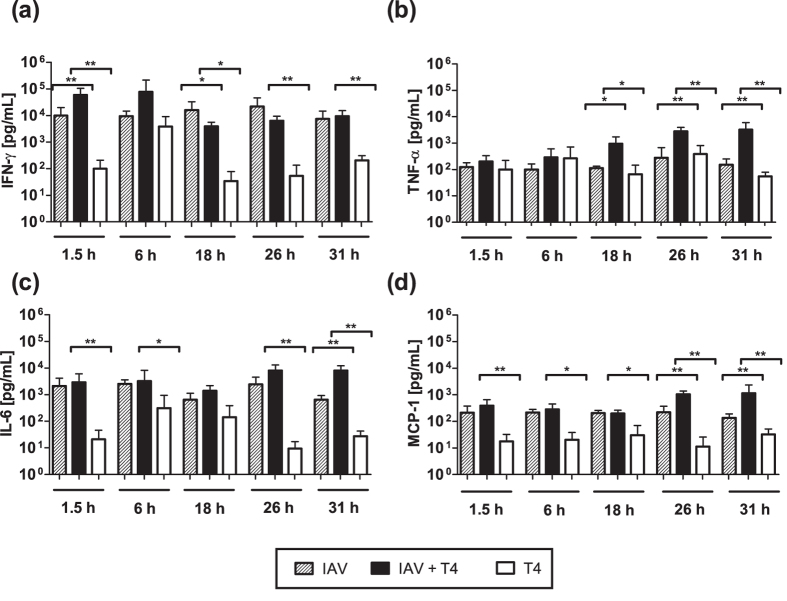
Pro-inflammatory cytokine profiles of the BAL of coinfected and single T4 infected mice. Protein concentrations of (**a**) IFN-*γ*, (**b**) TNF-*α*, (**c**) IL-6 and (**d**) MCP-1 were determined in the BAL fluid at the indicated time points after secondary T4 infection on day 7 post IAV or single T4 infection on day 7 post PBS treatment. Raw data can be found in the Supplementary Fig. S2. Statistical analysis was performed using the Mann-Whitney test. Asterisks indicate significant differences between single and coinfected mice: *p < 0.05; **p < 0.01.

In contrast to previous animal studies that often focused on a single time point post secondary infection^11^,^12^, we assessed the early kinetics of pro-inflammatory cytokines in the respiratory tract. For this purpose, we determined IFN-*γ*, TNF-*α*, IL-6, Monocyte Chemoattractant Protein-1 (MCP-1), Interferon-*β* (IFN-*β*), Interleukin 22 (IL-22) and Granulocyte-Macrophage Colony-Stimulating Factor (GM-CSF) protein concentrations in the BAL fluid of single and coinfected mice. A time-dependent significant increase in the protein concentrations of IFN-*γ*, TNF-*α*, and IL-6 was observed in the coinfected animals when compared to the single T4 infected animals (Fig. 4).

Bacterial infection alone led to a transient increase in IFN-*γ* at 6 hpi and IL-6 at 6 hpi and 18 hpi, which hardly reached the elevated IFN-*γ* and IL-6 levels detected in the IAV-infected group at all time points analyzed (Fig. 4). For IFN-*γ*, coinfection led to a further increase early at 1.5 hpi and 6 hpi compared to the single IAV infection whereas the levels remained constant compared to the underlying IAV infection for the later time points and a significant increase was only observed when compared to the single T4 infection. Overshooting IL-6 responses in the coinfected mice were detected at 26 hpi and 31 hpi compared to the single T4 infection. At the same time, TNF-*α* levels hardly changed between mice infected with IAV alone and T4 alone but were increasingly and significantly elevated in coinfected mice from 18 hpi on. This late excess in IL-6 and TNF-*α* compared to both the single IAV and T4 infected groups indicated a steady induction of these two pro-inflammatory cytokines in the coinfected animals (Fig. 4b and c). Of note, the chemokine MCP-1 was also significantly increased in the IAV+T4 group compared to the single T4 infected group and marginally increased to the IAV only group at 26 hpi and 31 hpi (Fig. 4d). The protein concentrations of the other inflammatory mediators did not show significant changes between the groups at all time points (see Supplementary Fig. S3). Note that different studies using C57BL/6 J mice reported the baseline levels of the mediators studied here to be significantly lower than what is detected following IAV infection^12^,^37^,^38^.

### Decisive role of IFN-*γ* in bacterial outgrowth

To dissect the temporal contribution of the measured pro-inflammatory cytokines in preventing bacterial clearance, mathematical models fitted from single *S. pneumoniae* infection were challenged in order to assess the effects of pro-inflammatory responses on bacterial lung titers. Hence, we adopted the best model for single *S. pneumoniae* infection (model D2 from the Supplementary 2) and challenged the mathematical term representing bacterial clearance (*c*_*b*_*B*) with different functions (*c*_*b*_*f*_*x*_*B*) to evaluate which of the pro-inflammatory cytokines or their combinations provided the best fit to the bacterial burden detected in the coinfected mice. A brief list of mathematical models tested is presented in Table 1 and a complete version with estimated parameters and parameter uncertainty analysis is shown in Supplementary 3 and 4, respectively.

Considering the AICc scores, criterion of small differences (less than 2 units) were not significant (see Materials and Methods), the best group of models was M3 and M7 (Table 1). The common component of these two models is the IFN-*γ kinetic* and, remarkably, a mechanism only based on the IFN-*γ* response (M3) provided a better fit than mechanisms based on only TNF-*α* (M4) or IL-6 (M5) even though a more conservative AICc criterion is considered (*e.g* ≤10). Additionally, different models presented in the Supplementary 3 (M6, M7, M8 and M9) that also had IFN-*γ* dynamics involved in the impairment function *f*_*x*_ scored closely to M3. In agreement with previous work^12^, our model selection process dissected IFN-*γ* as a key and sufficient modulator in the impairment of bacterial clearance.

### The IL-6 response contributes to the impairment of bacterial clearance in a temporal manner

Both the models M6 and M7 suggested that IL-6 may contribute to impairing bacterial clearance during coinfection. However, the AICc for the model M5 pointed out that it may not be as crucial as IFN-*γ* for impairing bacterial clearance. However, it could be deduced that IL-6 is involved in the majority of the best models (*e.g* M6, M7, M8 and M9), indicating a time-dependent detrimental role in curtailing bacterial outgrowth.

**Figure 5:**
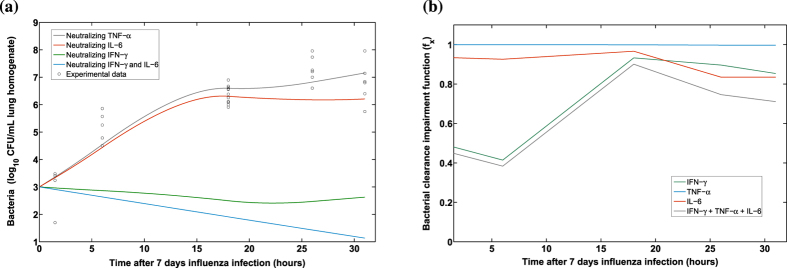
Simulations for the coinfection model M6. *In silico* neutralizations of the different pro-inflammatory responses in model M6 are presented in the panel (**a**). The time-dependent contributions of pro-inflammatory cytokines to the impairment of bacterial clearance by the function *f*_*x*_ are depicted in the panel (**b**). When the mathematical function *f*_*x*_ is 1 means that there is no impairment to the bacterial clearance.

To disentangle the time-dependent roles of significant pro-inflammatory cytokines, we selected the model M6 to perform *in silico* experiments. Simulation results suggested that a single neutralization of IFN-*γ* directly modulates the bacterial clearance. In contrast, neutralization of IL-6 or TNF-*α* did not present a clear role in impairing bacterial clearance (Fig. 5a). Interestingly, when the IFN-*γ* response was neutralized in the model, simulations suggested that the IL-6 response may increase the duration of bacterial colonization in the lungs (Fig. 5a). This possibly explains the experimental observation that coinfected mice still presented a marginal increase in the bacterial burden and degree of lethality even after *in vivo* IFN-*γ* blockade^12^.

To weigh the inhibitory effects of different pro-inflammatory cytokines on bacterial clearance, we simulated the time evolution of the bacterial clearance impairment function (*f*_*x*_) for the different pro-inflammatory cytokines using the model M6. Indeed, the main modulator early after *S. pneumoniae* coinfection was IFN-*γ*. However, after 18 hours the detrimental effects of IL-6 on the bacterial clearance became more apparent (Fig. 5b). In contrast, model selection procedures and numerical simulations did not support that TNF-*α* kinetics contributed to the impairment of bacterial clearance, yielding one of the worst models (Table 1). These *in silico* results were supported by other model structures (M7, M8 and M9 at Supplementary 3) and parameter uncertainty studies (Supplementary 4).

### Impairment of bacterial clearance by direct IAV kinetics is not supported

Viral titers in the single IAV infected group remained stable at day 7 post the viral infection, i.e 1.5 and 6 hpi post secondary challenge, and the viral load then declined from 26 hpi (see Supplementary Fig. S4b). In accordance with previous experimental observations by Smith *et al*.^33^, viral titers of the coinfected group showed a marginal, however not statistically significant viral rebound at 31 hpi, as the coinfected mice yielded higher viral loads than the single infected mice.

The modelling work by Smith *et al*.^33^ tested the hypothesis that influenza infection modulates the bacterial clearance by AM phagocytosis. However, their model did not include aspects of host defense^33^. As a comparison, we considered the inhibitory function proposed by Smith *et al*.^33^ using the kinetics of the viral load detected in our experiments (M10 at Supplementary 3). Model selection procedures however did not support that a time-dependent modulation by IAV kinetics contributes to the impairment of bacterial clearance.

## Discussion

The threat of newly emerging pandemic IAV strains together with the increasing prevalence of antibiotic-resistant bacterial pathogens underline the need for a complete understanding of the mechanisms for secondary pneumonia. Recently, a substantial number of studies have uncovered several mechanisms through which IAV compromises efficient anti-bacterial defense. These different contributing factors for the viral-bacterial synergism highlight the multifactorial nature of copathogenesis.

At times however, the identified mechanisms are contradictory, *e.g* both de-sensitization and hyper-sensitization have been reported to favor bacterial infection post influenza infection^10^,^13^,^39^. Nevertheless, a common observation is an altered pro-inflammatory cytokine response to the bacterial infection if preceded by influenza and many studies have attributed the altered lung cytokine milieu to be sufficient to skew host susceptibility to severe secondary pneumococcal infection^11^,^12^,^40^.

The relative contribution of the different identified players however has not been addressed comprehensively to date. Although murine experiments are crucial to advance our knowledge of mechanisms which underlie virus-bacteria interactions, examining all different factors in detail is extremely difficult work. In this study, we combined an *in vivo* experimental approach and mathematical modelling to dissect the contributions of some of the circulating hypotheses proposed in driving bacterial outgrowth in IAV pre-infected hosts with the main focus on pro-inflammatory cytokines.

Unlike described by previous reports^10^,^11^,^41^, in our experiments the inflammatory responses to secondary pneumococcal infection were not dampened but instead heightened when compared to the single bacterial infection. Through mathematical modelling we identified a definite role for IFN-*γ* in impairing anti-pneumococcal clearance leading to outgrowth and systemic spread. This was in line with the finding that IFN-*γ* released during IAV-infection suppresses alveolar macrophage phagocytosis and increases oxidative radicals by downregulating their expression of the scavenger receptor MARCO in a state of coinfection, favoring bacterial outgrowth^10^,^12^,^39^. As *in vivo* neutralization of IFN-*γ* failed to fully alleviate bacterial outgrowth following coinfection with IAV and *S. pneumoniae*, other mediators are most likely to also contribute to the strongly impaired bacterial clearance^12^.

In accordance with other possible key players during coinfection, we found in our murine experiments significantly higher concentrations of IL-6 and TNF-*α* during coinfection compared to the single infections^13^,^40^. The exact role of these cytokines in the pathogenesis of coinfections still remains debatable. Here, our *in silico* studies suggest that the inhibitory effect of IL-6 is both concentration and time-dependent but not as conclusive for bacterial outgrowth as IFN-*γ*. Early after pneumococcal infection, IL-6 is likely to play a predominantly protective role due to its immune-regulatory function in the feedback circuit of cytokines. Indeed, it was reported that IL-6 KO mice displayed a significantly enhanced susceptibility to *S. pneumoniae* infection with elevated concentrations of TNF-*α*, IL1-*β* and IFN-*γ* in the lungs in comparison to wild-type mice^42^. On the other hand, due to its potent pro-inflammatory functions, IL-6 is also known to be a marker for disease severity in pneumococcal infections^43^. Importantly, our finding that increased amounts of IL-6 predominantly impair bacterial clearance in synergism with the IAV-dependent IFN-*γ* present in the coinfected lung reflects how alterations in the inflammatory status affect host susceptibility in a dynamic temporal manner.

Interestingly, we observed that TNF-*α* showed the least contribution in impairing bacterial clearance following influenza. This was well in line with previous findings describing that TNF-*α* neutralization elevated mortality during single *S. pneumoniae* infection and IAV coinfections suggesting more of a protective role for TNF-*α*^44^,^45^. However, TNF-*α* is in contrast known to induce apoptosis of various pulmonary cells and to disrupt the epithelial barrier integrity and therefore TNF-*α* blockade ameliorates pulmonary immunopathology in single IAV infected animals^46^,^47^,^48^. Nevertheless, in our study, effects such as the detrimental contribution of immunopathology on *S. pneumoniae* outgrowth were not modelled. Therefore it is likely that an excessive production of TNF-*α* during coinfections may potentiate host tissue damage and thereby may still exert an indirect negative influence on bacterial clearance. Regarding the protein MCP-1, as this chemokine regulates migration and infiltration of AMs, we assumed that the downstream effects of MCP-1 were reflected in the AM dynamics (Fig. 3 and the model M1). However, in order to analyse the hypothesis that MCP-1 may directly play a role during coinfections, the model M12 in Supplementary 3 was tested. The AICc criterion did not rank MCP-1 kinetics as a key mechanism to describe the bacterial burden in coinfected animals.

Under homeostatic conditions, tightly regulated immune responses are co-ordinated by the action of pro- and anti-inflammatory cytokines and chemokines with the goal to clear pathogens and at the same time curtail immunopathology. By our experimental and mathematical modelling approach, we found that during coinfections this balance is lost due to the exaggerated amounts of pro-inflammatory cytokines causing a pathogenic effect on bacterial clearance. Notably, this finding was further supported by the results of challenging our mathematical models for the IAV-*S. pneumoniae* coinfection with the cytokine data from the single *S. pneumoniae* infection group, *in silico* results predicted bacterial clearance as in the single bacterial infection without further parameter fitting (Fig. S5).

Previous studies showed that the abundance of IFN-*γ* driven mainly by T cells enhances the susceptibility to secondary pneumococcal coinfection^12^. Nevertheless, IFN-*γ* plays a central role in host defence and immunity against single infections to IAV^49^ and *S. pneumoniae*^50^,^51^, pointing out an essential role for the recruited neutrophils in the production of IFN-*γ* in response to *S. pneumoniae*^50^,^51^. Furthermore, experiments in mice showed that deletion of IFN-*γ* resulted in a more severe impairment of lung function^49^. Therefore, further modelling and experimental efforts are needed to uncover the bifurcation point that shifts IFN-*γ* from benign to detrimental in coinfections.

In conclusion, by combining tailored experimental data and mathematical modelling our study suggested a strong detrimental effect of IFN-*γ* alone and in synergism with IL-6 but no conclusive pathogenic effect of IL-6 or TNF-*α* alone. *In silico* predictions pave the way for further murine experiments to prove or reject the advantage of a double IFN-*γ* and IL-6 neutralization approach. Ultimately, these findings correlate well with our previous results^31^ suggesting that the increased levels of pro-inflammatory cytokines (the “inflammaging” state), in particular IFN-*γ*, contribute to the reported impaired responses in people over 65 years of age. Thereby, IFN-*γ* plays a pivotal role in driving severe disease during primary IAV infection in the elderly as well as bacterial outgrowth during coinfections. Ultimately, such full understanding of the downstream effects of the altered inflammatory response in bacterial coinfection following IAV will be crucial when attempting to design future prophylactic and therapeutic interventions.

## Materials and Methods

### Mice

7–8 weeks old wildtype C57BL/6J female mice were purchased from Harlan Winkelmann (Borchen, Germany) and housed in specific pathogen-free conditions.

### Ethics Statement

All animal experiments were approved by the local ethical body “Niedersächsisches Landesamt für Verbraucherschutz und Lebensmittelsicherheit” (file number 33.9-42502-04-11/0443) and were conducted in conformity with the German animal welfare act and the European Communities Council Directive 2010/63/EU.

### Viral and bacterial pathogens

For viral infection experiments, the influenza A virus strain A/Puerto Rico/8/1934 (A/PR8/34) subtype H1N1 was used^34^. The virus was grown and stored as described previously in ref. 34. The tissue culture infectious dose (TCID_50_) was calculated using the Reed and Muench endpoint calculations. For bacterial infection experiments *S. pneumoniae* serotype 4, TIGR4 (ATCC BAA-334^TM^) was used. Bacteria were grown to mid-logarithmic phase in Todd-Hewitt yeast medium (THY; THB Sigma-Aldrich, Germany and Yeast extract, Roth, Germany) at 37 °C for the preparation of frozen stocks. Bacterial counts were determined by plating 10-fold serial dilutions on blood agar plates (BD Diagnostic Systems, Columbia Agar with 5% sheep blood) overnight for 16–18 h at 37 °C with 5% CO_2_. Before each infection, aliquots were thawed, centrifuged at 8000 rpm and resuspended in the desired amount of phosphate buffered saline (PBS, Gibco, UK) followed by plating of serial dilutions to confirm the infection dose.

### Infection experiments

Mice were sedated via intraperitoneal injection of ketamine/rompun (working concentration of 0.1 ml/10 g per mouse). For viral challenges, a sub-lethal dose of 0.31 TCID_50_ was administered intranasally in 25 *μ*l PBS. For bacterial challenges, mice were placed with their backs on an intubation slope, and an infectious dose of 1 × 10^6^ CFU in 25 *μ*l PBS was instilled at the end of their nasopharynx using a long flexible pipet tip. All the mice were monitored and scored for the following clinical symptoms: weight loss, mobility, posture, pilo-erection, respiration, response to stimuli and eye infection. Animals with a severe score of any one of the parameters or moderate scores for two to three parameters were euthanized.

### Determination of bacterial colony forming units in blood, bronchoalveolar lavage and lung tissue

Bacterial CFU were determined post-mortem by plating 10-fold serial dilutions of the blood, BAL and lung tissue homogenate. For the blood, 5 *μ*l of heart blood were collected and diluted in 45 *μ*l PBS. BAL samples were obtained by flushing the lung once with 1 mL PBS. Next, the lungs were perfused with PBS and excised. Whole lungs were homogenized in 1 mL PBS though a 100 *μ*m cell stainer (Corning Inc, USA). Serial dilutions of BAL and lung homogenates were prepared and 10 *μ*l were plated onto blood agar plates. Plates were incubated overnight at 37 °C and 5% CO_2_. For determination of CFU the colonies of each dilution were counted manually and calculated as CFU/mL.

### Quantification of viral load with qRT-PCR

Lungs were perfused with PBS and the RNA was extracted from lung tissue homogenates with the RNeasy mini kit (Qiagen, Germany) according to the manufacturer’s protocol. After extraction and purification, cDNA synthesis was performed with 100 ng RNA using the SuperScript III first-strand synthesis System (Invitrogen, USA). For cDNA quality control, a PCR of the housekeeping gene RPS9 (Eurofins MWG, Germany) was performed. Viral nucleoprotein copy numbers were quantified by RT-PCR on a Light cycler (Roche) using a plasmid standard with defined NP copy numbers. The following primers were used: rps9 5′CTGGACGAGGGCAAGATGAAGC and 3′TGACGTTGGCGGATGAGCACA; np 5′GAGGGGTGAGAATGGACGAAAAAC and 3′CAGGCAGGCAGGCAGGACTT.

### Flow cytometry for alveolar macrophages

Single cell suspensions of perfused excised lungs were prepared by enzymatic digestion for 45 minutes at 37 °C with Iscove’s Modified Dulbecco’s Medium (IMDM) containing GlutaMax-1 (Gibco, USA) supplemented with 5% fetal bovine serum (FBS; PanBiotech, Germany), 0.2 mg/ml collagenase D (Roche, Germany) and 1 mg/ml DNAse (Sigma-Aldrich, Germany). The enzymatic reaction was stopped with 5 mM EDTA and cell suspensions were filtered through a 100 *μ*m strainer.

Cells were pelleted by centrifugation and resuspended in LIVE/DEAD^®^ fixable blue stain (ThermoFisher, USA) and anti-mouse CD16/CD32 antibody (purified; BioLegend, USA) as Fc-block following erythrocyte lysis. Cell surface marker staining was performed using anti-mouse CD11b (BioLegend, USA) and anti-mouse F4/80 (BioLegend, USA). All samples were acquired on a BD LSRII Fortessa instrument with FACS DIVA software (BD) and analyzed using FlowJo software (Tree Star). Following debris and dead cell exclusion, AMs were identified as SSChigh, FSChigh, CD11b- and F4/80+ autofluorescent cells. In separate analyses the AM phenotype of the cells identified by this strategy was confirmed by CD11c staining. Absolute cell counts were calculated from the manually counted overall cell number in a sample and the population frequency assessed by flow cytometry.

### Cytokine measurement

The protein concentration of the cytokines IFN-*γ*, TNF-*α*, IL-6, IFN-*β*, IL-22 and the chemokines MCP-1 and GM-CSF was measured with a customized Mouse LegendPlex™ kit (BioLegend, USA) according to the manufacturers protocol. All samples were acquired on a BD LSRII Fortessa with FACS DIVA software (BD) and analyzed with the provided LegendPlex™ Data Analysis Software (BioLegend, USA).

### Mathematical Models

This work aimed to disentangle the mechanisms modulated by IAV that can contribute to the colonization of *S. pneumoniae*. Although, we recognized that bacterial infection may enhance viral release from infected cells^33^, these interactions were outside of the aim of the work proposed here. Therefore, we modelled the *S. pneumoniae* infection (*B*) with the following ordinary differential equation (ODE):


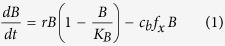


where *r* is the bacterial proliferation rate with a maximum carrying capacity *K*_*B*_. Phagocytosis of the bacteria is considered by the multiplicative term *c*_*b*_*f*_*x*_, where *c*_*b*_ is the constant clearance rate and the term *f*_*x*_ is the mathematical function which served to evaluate different hypotheses.

The previous work by Smith *et al*.^33^ proposed and tested that IAV induces phenotypic changes in AM. However, the work in ref. 33 assumed the restrictive condition that the phagocytosis rate *c*_*b*_ is impaired by the viral load (*V*(*t*)). This hypothesis was written with the term 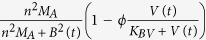 where the values for the uptake of bacteria by AM were expressed by *n* and the number of AM(M_A_) was chosen to be equal to the quasi steady state value 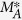 33. The reduction of the bacterial clearance due to the viral effects was included by the saturation function *ϕV*(*t*)/(*K*_*BV*_ + *V*(*t*)), where *ϕ* is the maximal reduction of the phagocytosis rate and *K*_*BV*_ is the half-saturation constant.

In order to evaluate the AM dynamics, our model uses the AM experimental data to build a piecewise linear function to determine the model term *M*_*A*_(*t*). Additionally, we examined the hypothesis that AM depletion by IAV is a sufficient mechanism to facilitate bacterial outgrowth. This is represented by a direct input of AMs data in equation (1).

Beyond the work of ref. 33, different functions (*f*_*x*_) were tested to dissect the contributions of pro-inflammatory cytokines (IFN-*γ*, TNF-*α*, and IL-6) to modulate the bacterial clearance. To this end, we used the experimental data for the different cytokines to build piecewise linear functions to dynamically determine the equation (1). We opted for this approach instead of mechanistic modelling with an ODE for each cytokine for different reasons. First, our main aim was not to quantify dynamics but to determine the contribution of important pro-inflammatory cytokines in promoting bacterial colonization. Second, modelling with an ODE for each cytokine may not improve the model selection process but only increase the complexity of mathematical models and parameter fitting procedures. Finally, our experimental data were measured frequently enough allowing us to use the data as an input in the equation (1).

To evaluate if IAV infection modulates a specific pro-inflammatory cytokine *X*(*t*), which subsequently facilitates bacterial colonization, we considered the saturating term 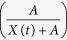 with *A* as the “half-saturation constant”. Different mathematical terms and other term combinations to dissect the pro-inflammatory effects responsible to impair bacterial clearance are summarized in Table 1 and Supplementary 3.

### Parameter Fitting

Note that the purpose of our *in silico* work was not biological quantification of the experiments but using a model selection process to provide mechanistic insights into coinfection. Thus, in this case, finding a working set of parameter values deemed sufficient. Nevertheless, for good practice procedures, we checked identifiability and parameter uncertainty expanded in the Supplementary 4.

To focus on the mechanisms that promote *S. pneumoniae* colonization, we fixed the growth rate (r = 1.13 h^−1^) and the carrying capacity (*K*_*B*_ = 2.3 × 10^8^ CFU/ml) corresponding to single *S. pneumoniae* infection from previous works^20^. Using similar reasoning as Smith *et al*.^20^, we assumed that only a proportion of the bacterial inoculum may reach the lung since some bacteria can be removed by mucocilliary mechanisms. Thus, we considered 1000 CFU/mL *S. pneumoniae* as inoculum to fit the model parameters in model (1). We would like to remark different assumptions of parameters and inoculation will only rescale the fitted parameters, but not the mechanistic insights from the model selection procedures.

Other model parameters were obtained minimizing the residual sum of squares (RSS) between the model output and the experimental measurement, both on log scales. ODEs were solved in Matlab software using the ode45 solver. The minimization of RSS was performed using different optimization solvers, including both deterministic and stochastic methods. However, the Differential Evolution algorithm (DE), a global optimization algorithm, was selected to avoid relying on any initial parameter guesses and producing more robust results than other tested methods^52^,^53^. In separate form, model fitting was performed to all the models summarized in Table 1 for the single infection (T4) and the IAV+T4 coinfection starting at day 7 post IAV infection respectively.

### Model Selection

The Akaike information criterion (AIC) was used to compare the goodness-of-fit of models that represent different hypotheses^54^. A lower AIC value means that a given model describes the data better than other models with higher values. Absolute difference in AIC greater than two units indicates stronger evidence for one model over the other^54^. For a small number of data points, the corrected Akaike information criterion (AICc) has the form:


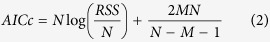


where *N* is the number of data points, *M* is the number of unknown parameters and *RSS* is the residual sum of squares obtained from the fitting routine.

### Parameter uncertainty

Bootstrapping is a statistical method for assigning measures of accuracy to estimates^55^,^56^. Due to the large variability of viral and bacterial infections, bootstrapping methods have been applied to different viral infectious diseases^53^,^57^. Here, the non-parametric bootstrap was considered. Of note, instead of using all replications at each time point, three replications at each time point were randomly selected and used to generate a new data set. Note that this procedure was based exclusively on the observed measurement data. For each repetition, the model parameters were refitted to obtain the corresponding parameter distribution. The 95% Confidence Interval (CI) of parameter estimates was computed using the outcome of the bootstrap method^56^. For each parameter, the 2.5% and 97.5% quantiles of the estimates were used to form the 95% CI.

### Parameter identifiability

A relevant aspect to verify in quantifying mechanisms is whether model parameters are identifiable based on the measurements of output variables^58^,^59^,^60^,^61^. A system that is algebraically identifiable may still be practically non-identifiable if the amount and quality of the measurements is insufficient and the data shows large deviations. The computational approach proposed in ref. 62 exploits the profile likelihood to determine identifiability and was considered here. This method is able to detect both structurally and practically non-identifiable parameters. Briefly, the idea behind this approach is to explore the parameter space for each parameter *θ*_*i*_ by re-optimizing the RSS with respect to all other parameters *θ*_*j*≠*i*_. The main task is to detect directions where the likelihood flattens out^62^. The resulting profiles are plotted versus each parameter range with the respective 95% CI to assess the parameter identifiability.

### Statistical Analysis

All statistical analyses were performed by the Mann-Whitney test using the Graph Pad Prism software (Graph Pad Software, La Jolla/USA).

## Additional Information

**How to cite this article**: Duvigneau, S. *et al*. Hierarchical effects of pro-inflammatory cytokines on the post-influenza susceptibility to pneumococcal coinfection. *Sci. Rep.* **6**, 37045; doi: 10.1038/srep37045 (2016).

**Publisher's note:** Springer Nature remains neutral with regard to jurisdictional claims in published maps and institutional affiliations.

## Supplementary Material

Supplementary Information

## Figures and Tables

**Table 1:** Selected list of the coinfection models to test different hypotheses that facilitate *S. pneumoniae* colonization.

No	Hypothesis	*f*_*x*_	Parameters	RSS	AICc
M1	AM number dynamics *M*_*A*_(*t*) are sufficient to facilitate bacterial outgrowth^36^.	*M*_*A*_(*t*)	47.74	12.27	*c_b_B*
M2	Single change in bacterial clearance rate is enough to explain bacterial outgrowth.	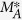	*c_b_B*	21.84	−15.87
M3	IFN-*γ* responses alone can impair bacterial clearance facilitating bacterial colonization^12^.	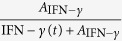	A_IFN-*γ*_(*t*)	16.13	**−26.78**
M4	TNF-*α* responses alone can impair bacterial clearance facilitating bacterial colonization^63^.	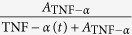	A_TNF-α_**	39.82	5.74
M5	IL-6 responses alone can impair bacterial clearance facilitating bacterial colonization^63^.	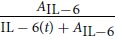	A_IL-6_	27.22	−7.94
M6	A synergistic effect of the IFN-*γ (X*_1_), IL-6 (*X*_2_), and TNF-*α (X*_3_) cytokine responses in facilitation of bacterial outgrowth.	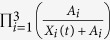	A_IFN-*γ*_, A_IL-6_ *A_*TNF–α*_*	14.57	−25.82
M7	A synergistic effect of the IFN-*γ (X*_1_) and IL-6 (*X*_2_) cytokine responses in facilitation of bacterial outgrowth.	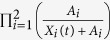	A_*IFN-*γ**_, A_*IL-6*_	14.57	**−28.21**

Best models based on AICc difference lower than 2 units are in bold. A complete model list with estimated parameters can be found in Supplementary 3.
