# Supplementary material for: Hierarchical effects of pro-inflammatory cytokines on the post-influenza susceptibility to pneumococcal coinfection
Source: Sci Rep. 2016 Nov 22;6:37045. doi: 10.1038/srep37045 (PMC5181841; doi:10.1038/srep37045)
Supplement: Supplementary Information [file srep37045-s1.pdf]

Supplementary Information

Hierarchical effects of pro-inflammatory cytokines on the post-influenza susceptibility to pneumococcal coinfection

Stefanie Duvigneau[1,2,3,4,+], Niharika Sharma-Chawla[1,3,+], Alessandro Boianelli[2], Sabine Stegemann-Koniszewski[1,3], Van Kinh Nguyen[2], Dunja Bruder[1,3,†], Esteban A. Hernandez-Vargas[2,†,*]

1 Infection Immunology Group, Institute of Medical Microbiology, Disease Prevention and Control, Otto-von-Guericke University Magdeburg, Germany.

2 Systems Medicine of Infectious Disease Group, Department of Systems Immunology and Braunschweig Integrated Centre of Systems Biology, Helmholtz Centre for Infection Research, Braunschweig, Germany

3 Immune Regulation Group, Helmholtz Centre for Infection Research, Braunschweig, Germany

4 Chair for Automation/Modeling, Institute for Automation Engineering, Otto-von-Guericke University Magdeburg, Germany

+ Contributed equally to this work.

† Contributed equally last author.

* Corresponding author: Esteban.Vargas@helmholtz-hzi.de

1 Supplemental Experimental Data Information

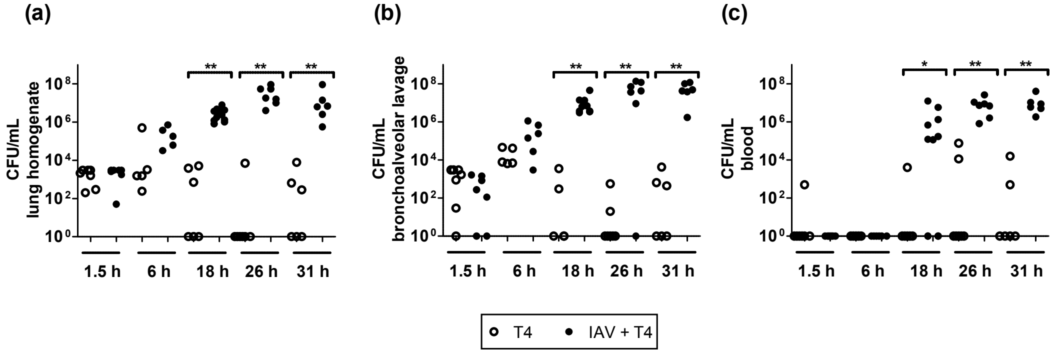

Figure S1. **CFU counts in lung, BAL, and blood of single T4 infected and coinfected animals (IAV+T4) at the indicated time points.** Asterisks indicate significant differences between single and coinfected mouse groups: * $p<0.05$; ** $p<0.01$.

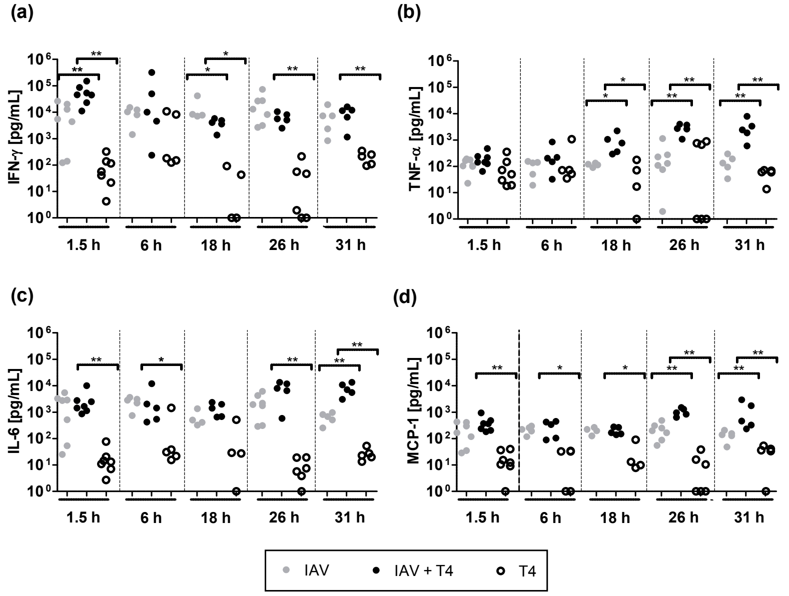

Figure S2. **Time-course kinetics of the protein concentrations of IFN-γ, TNF-α, IL-6 and MCP-1 in the BAL of coinfected (IAV+T4), single IAV or T4 infected mice.** Asterisks indicate significant differences between single and coinfected mouse groups: * $p<0.05$; ** $p<0.01$.

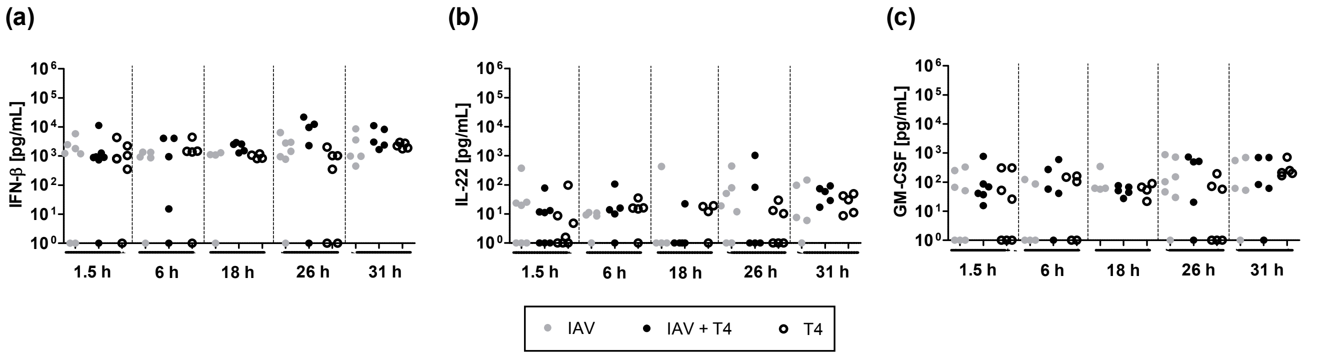

Figure S3. **Time-course kinetics of the protein concentrations of IFN-β, IL-22 and GM-CSF in the BAL of coinfected (IAV+T4), single IAV or T4 infected mice.**

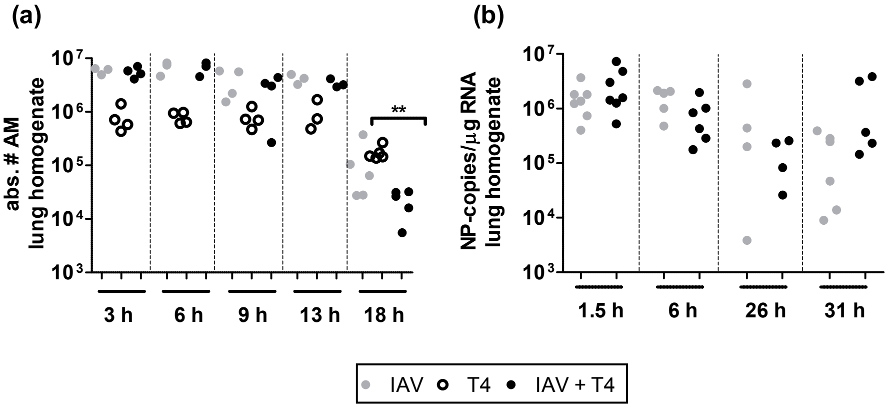

Figure S4. **Absolute numbers of AMs and viral titer data.** Asterisks indicate significant differences between single and coinfected mouse groups: ** p<0.01.

2 Models for single *S. pneumoniae* infection

Table S1. The role of alveolar macrophages (AM) during single *S. pneumoniae* infection. The function f_x will serve to test different hypotheses for bacterial clearance in equation (1).

No	Hypothesis	Assumption	f_x	Estimated Parameters	RSS	AICc
D1	AM kinetics ($M_A(t)$) modulate the bacterial clearance during single *S. pneumoniae* infection.	The term $M_A(t)$ represents a piecewise linear function.	$M_A(t)$	$c_b = 2.21 \times 10^{-6}$	143.39	48.49
D2	Constant AM counts (M_A^*) are sufficient to represent bacterial clearance in single *S. pneumoniae* infection.	The fate decision of not growing takes place in the first 18 hours post *S. pneumoniae* infection, during this time window M_A is approximately 7.8×10^5.	M_A^*	$c_b = 1.64 \times 10^{-6}$<br>$M_A c_b = 1.28$	62.10	**24.23**
D3	Small numbers of AM are necessary to clear *S. pneumoniae*.	The bacterial clearance takes place by small numbers of AM, the smallest experimental amount of M_A reported was approximately 1.73×10^5.	M_A^*	$c_b = 7.35 \times 10^{-6}$<br>$M_A c_b = 1.28$	62.10	**24.23**
D4	AM clearance decreases with the bacterial colony size[33].	The AM steady state is assumed to be 10^6[33].	$\frac{n^2 M_A^*}{n^2 M_A^* + B^2}$	$c_b = 1.69 \times 10^{-6}$<br>$n = 3$	61.07	**26.05**

[a] Following parameters were fixed: $B_0 = 10^3$, $r = 1.13$, and $K_b = 2.3 \times 10^8$.

[b] Best models based on AICc difference lower than 2 units are in bold.

3 Models for coinfection

Table S2. Model selection to dissect pro-inflammatory cytokine responses altered by IAV that can inhibit bacterial clearance.

Model	f_x	Estimated Parameters	RSS	AICc
M1	$M_A(t)$	$c_b = 1.64 \times 10^{-7}$	47.74	12.27
M2	$M_A^* = 7.8 \times 10^5$	$c_b = 9.18 \times 10^{-7}$<br>$M_A^* c_b = 0.72$	21.84	-15.87
M3	$\frac{A_{\text{IFN-}\gamma}}{\text{IFN-}\gamma(t)+A_{\text{IFN-}\gamma}}$	$A_{\text{IFN-}\gamma} = 4.03 \times 10^4$	16.13	**-26.78**
M4	$\frac{A_{\text{TNF-}\alpha}}{\text{TNF-}\alpha(t)+A_{\text{TNF-}\alpha}}$	$A_{\text{TNF-}\alpha} = 448.17$	39.82	5.74
M5	$\frac{A_{\text{IL-6}}}{\text{IL-6}(t)+A_{\text{IL-6}}}$	$A_{\text{IL-6}} = 3.31 \times 10^3$	27.22	-7.94
M6	$\prod_{i=1}^{3}\left(\frac{A_i}{X_i(t)+A_i}\right)$	$A_{\text{IFN-}\gamma} = 5.46 \times 10^4$<br>$A_{\text{IL-6}} = 4.05 \times 10^4$<br>$A_{\text{TNF-}\alpha} = 1.10 \times 10^6$	14.57	-25.82
M7	$\prod_{i=1}^{2}\left(\frac{A_i}{X_i(t)+A_i}\right)$	$A_{\text{IFN-}\gamma} = 5.46 \times 10^4$<br>$A_{\text{IL-6}} = 4.05 \times 10^4$	14.57	**-28.21**
M8	$\frac{1}{\sum_{i=1}^{3} A_i X_i(t)+1}$	$A_{\text{IFN-}\gamma} = 1.94 \times 10^{-5}$<br>$A_{\text{IL-6}} = 2.62 \times 10^{-5}$<br>$A_{\text{TNF-}\alpha} = 2.38 \times 10^{-6}$	14.55	-25.85
M9	$\frac{1}{\sum_{i=1}^{2} A_i X_i(t)+1}$	$A_{\text{IFN-}\gamma} = 1.94 \times 10^{-5}$<br>$A_{\text{IL-6}} = 2.62 \times 10^{-5}$	14.55	**-28.23**
M10	$\frac{n^2 M_A^*}{n^2 M_A^* + B^2}\left(1-\phi\frac{V}{K_{BV}+V}\right)$	$\phi = 0.30$<br>$K_{BV} = 100$	74.13	30.36
M11	$\sum_{i=1}^{2}\left(\frac{A_i}{X_i(t)+A_i}\right)$	$A_{\text{IFN-}\gamma} = 1.02 \times 10^4$<br>$A_{\text{IL-6}} = 1.04 \times 10^3$	15.71	-25.48
M12	$\frac{A_{\text{MCP-1}}}{\text{MCP-1}(t)+A_{\text{MCP-1}}}$	$A_{\text{MCP-1}} = 271.82$	30.99	-3.33

[a] Following parameters were fixed: $B_0 = 10^3$, $r = 1.13$, and $K_b = 2.3 \times 10^8$. The bacterial clearance value $c_b = 1.28$ is taken from the single *S. pneumoniae* infection (model D3 at Supplementary 2), this includes also the number of macrophages.

[b] Best models based on AICc difference lower than 2 units are in bold.

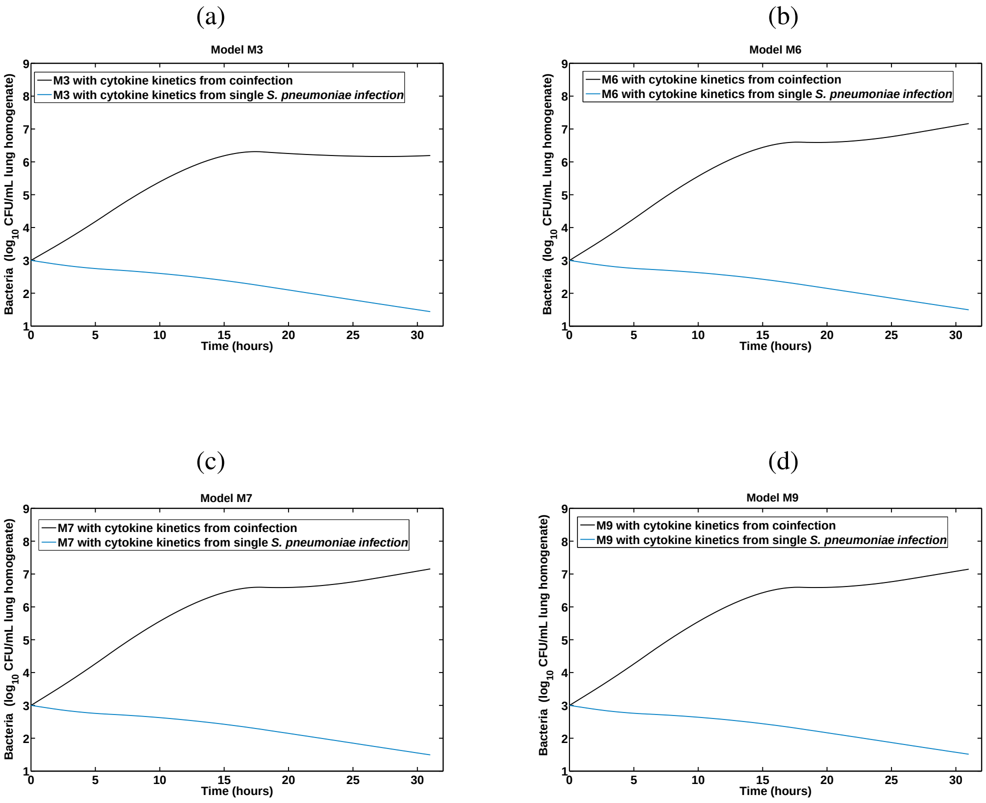

Figure S5. Challenging models for coinfection with the cytokine kinetics from the single *S. pneumoniae* infection. Models fitted with coinfected data (black lines) were challenged with the cytokine data from the single *S. pneumoniae* infection to predict the bacterial clearance (blue lines). Although models were fitted only with cytokine data from coinfection (black line), simulation results reveal that models can accurately determine the contribution and levels of pro-inflammatory cytokines (blue line), showing the possibility of the models to go beyond selection model procedures.

4 Parameter Uncertainty Studies

(a)

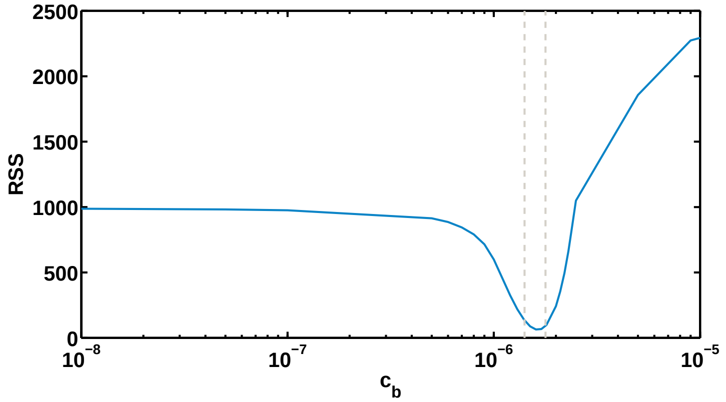

(b)

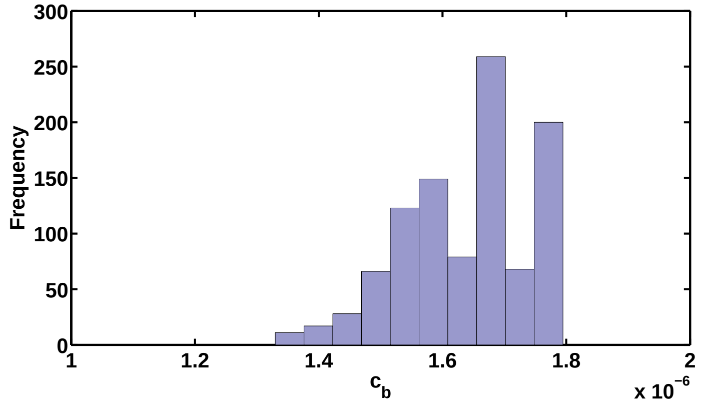

Model	f_x	Parameter	Best fit to all data	95% confidence interval	Lower and upper bound used for fitting procedures
D2	$M_A^* = 7.8 \times 10^5$	c_b	$1.64{\times}10^{-6}$	$[1.41\ 1.78]{\times}10^{-6}$	$[10^{-8}\ 10^{-4}]$

Figure S6. **Parameter uncertainty analysis for model D2.** (a) Profile likelihood for c_b. Vertical dashed lines represent the 95% confidence intervals. (b) Histogram of bootstrapping for parameter c_b in single *S. pneumoniae* infection. Bootstrapping was performed with 1000 samples.

(a)

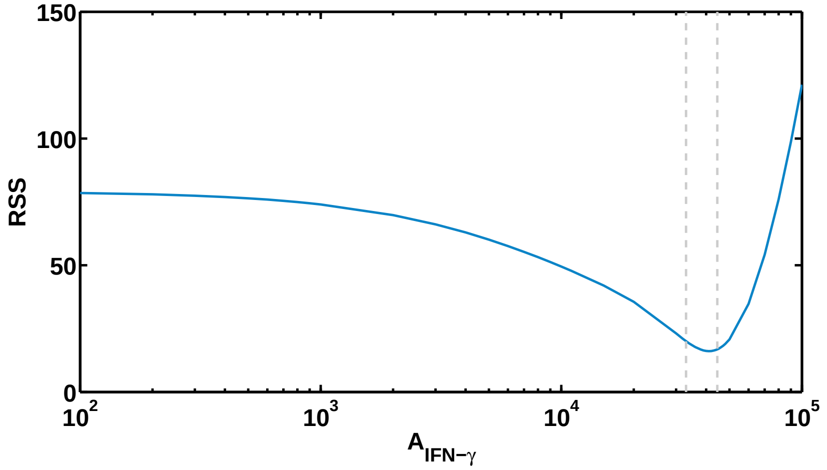

(b)

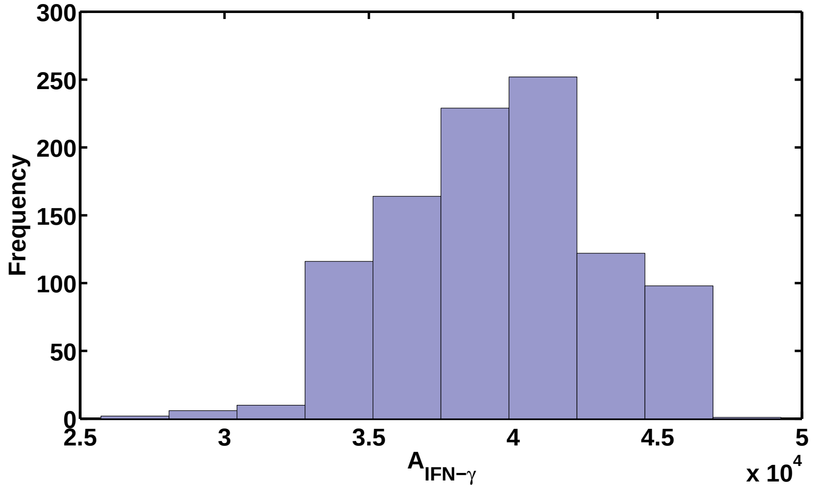

Model	f_x	Parameter	Best fit to all data	95% confidence interval	Lower and upper bound used for fitting procedures
M3	$\frac{A_{\text{IFN-}\gamma}}{\text{IFN-}\gamma(t)+A_{\text{IFN-}\gamma}}$	$A_{\text{IFN-}\gamma}$	4.03×10^4	$[3.30\ 4.45]\times10^4$	$[10^2\ 10^6]$

Figure S7. Parameter uncertainty analysis for model M3. (a) Profile likelihood for $A_{\text{IFN-}\gamma}$. Vertical dashed lines represent the 95% confidence intervals. (b) Histogram of bootstrapping for parameter $A_{\text{IFN-}\gamma}$ in single *S. pneumoniae* infection. Bootstrapping was performed with 1000 samples.

(a)

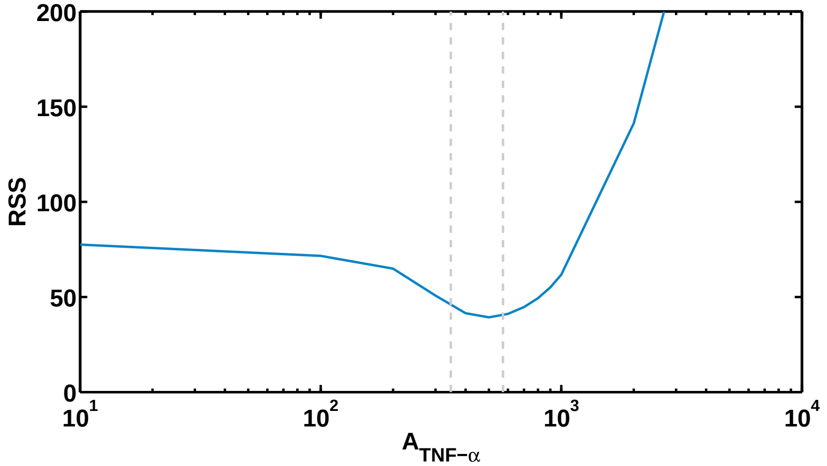

(b)

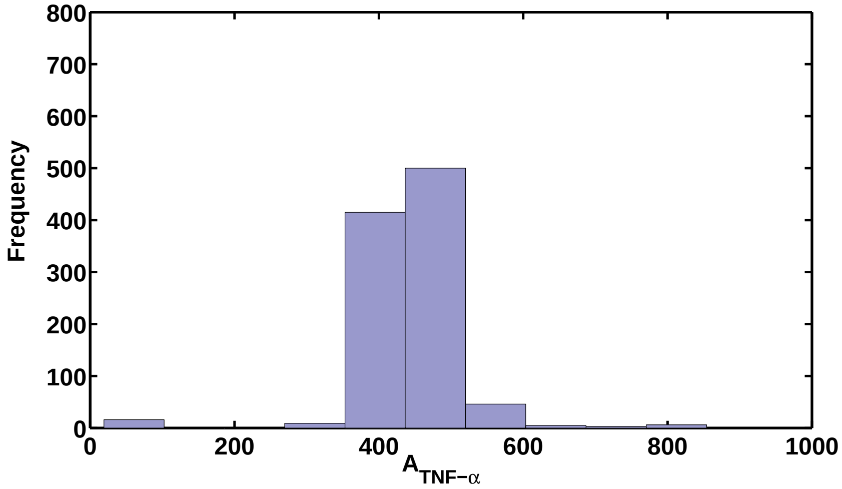

Model	f_x	Parameter	Best fit to all data	95% confidence interval	Lower and upper bound used for fitting procedures
M4	$\frac{A_{\text{TNF-}\alpha}}{\text{TNF-}\alpha(t)+A_{\text{TNF-}\alpha}}$	$A_{\text{TNF-}\alpha}$	448.17	[347.23 572.49]	[1 10^4]

Figure S8. Parameter uncertainty analysis for model M4. (a) Profile likelihood for $A_{\text{TNF-}\alpha}$. Vertical dashed lines represent the 95% confidence intervals. (b) Histogram of bootstrapping for parameter $A_{\text{TNF-}\alpha}$ in single *S. pneumoniae* infection. Bootstrapping was performed with 1000 samples.

(a)

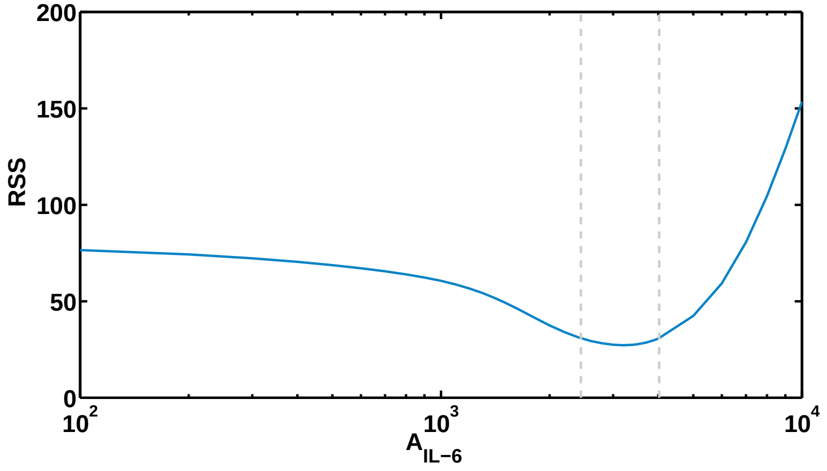

(b)

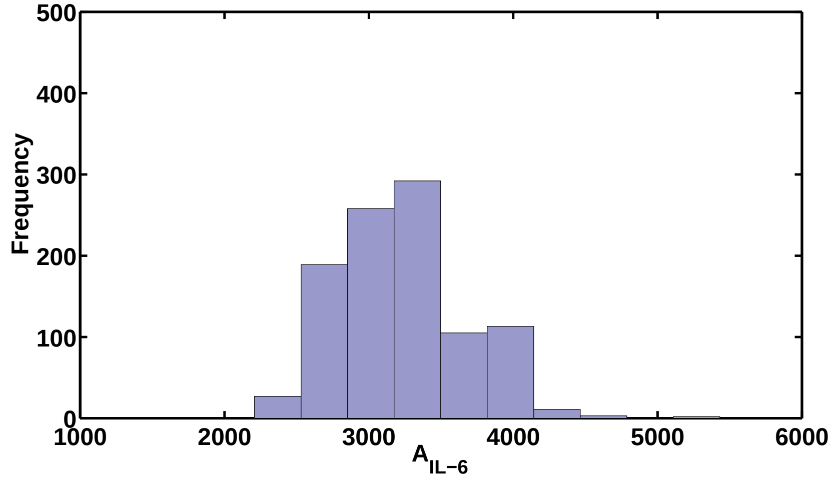

Model	f_x	Parameter	Best fit to all data	95% confidence interval	Lower and upper bound used for fitting procedures
M5	$\frac{A_{\text{IL-6}}}{\text{IL-6}(t)+A_{\text{IL-6}}}$	$A_{\text{IL-6}}$	3342.3	[2440.63 4023.9]	[1 10^4]

Figure S9. Parameter uncertainty analysis for model M5. (a) Profile likelihood for $A_{\text{IL-6}}$. Vertical dashed lines represent the 95% confidence intervals. (b) Histogram of bootstrapping for parameter $A_{\text{IL-6}}$ in single *S. pneumoniae* infection. Bootstrapping was performed with 1000 samples.

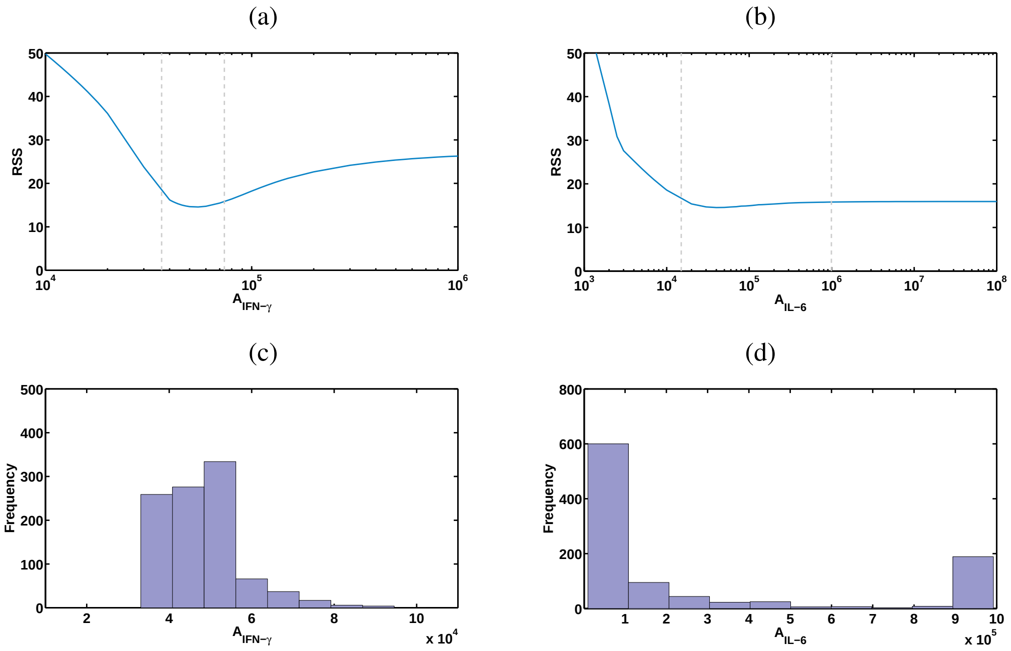

Model	f_x	Parameter	Best fit to all data	95% confidence interval	Lower and upper bound used for fitting procedures
M7	$\prod_{i=1}^{2}\left(\frac{A_i}{X_i(t)+A_i}\right)$	$A_{\text{IFN-}\gamma}$	5.46×10^4	$[3.66\ 7.37]\times 10^4$	$[10^3\ 10^6]$
		$A_{\text{IL-6}}$	4.05×10^4	$[0.15\ 9.92]\times 10^5$	$[10^3\ 10^6]$

Figure S10. Parameter uncertainty analysis for model M7. Panels (a) and (b) present the profile likelihood for $A_{\text{IFN-}\gamma}$ and $A_{\text{IL-6}}$ respectively. Vertical dashed lines represent the 95% confidence intervals. Panels (c) and (d) present the histograms of bootstrapping for parameter $A_{\text{IFN-}\gamma}$ and $A_{\text{IL-6}}$ respectively. Bootstrapping was performed with 1000 samples.
